# Obesity-associated cardiometabolic complications in polycystic ovary syndrome: The potential role of sodium-glucose cotransporter-2 inhibitors

**DOI:** 10.3389/fendo.2023.951099

**Published:** 2023-02-15

**Authors:** Jacob E. Pruett, Damian G. Romero, Licy L. Yanes Cardozo

**Affiliations:** ^1^ Department of Cell and Molecular Biology, University of Mississippi Medical Center, Jackson, MS, United States; ^2^ Mississippi Center of Excellence in Perinatal Research, University of Mississippi Medical Center, Jackson, MS, United States; ^3^ Women’s Health Research Center, University of Mississippi Medical Center, Jackson, MS, United States; ^4^ Cardiovascular-Renal Research Center, University of Mississippi Medical Center, Jackson, MS, United States; ^5^ Department of Medicine, University of Mississippi Medical Center, Jackson, MS, United States

**Keywords:** polycystic ovary syndrome (PCOS), obesity, SGLT 2 inhibitors, cardiometabolic complications, hypertension, insulin resistance, mitochondrial dysfunction, women’s health

## Abstract

Polycystic Ovary Syndrome (PCOS) is the most common endocrine disorder in reproductive-age women. PCOS is characterized by androgen excess, oligo/anovulation, and polycystic appearance of the ovaries. Women with PCOS have an increased prevalence of multiple cardiovascular risk factors such as insulin resistance, hypertension, renal injury, and obesity. Unfortunately, there is a lack of effective, evidence-based pharmacotherapeutics to target these cardiometabolic complications. Sodium-glucose cotransporter-2 (SGLT2) inhibitors provide cardiovascular protection in patients with and without type 2 diabetes mellitus. Although the exact mechanisms of how SGLT2 inhibitors confer cardiovascular protection remains unclear, numerous mechanistic hypotheses for this protection include modulation of the renin-angiotensin system and/or the sympathetic nervous system and improvement in mitochondrial function. Data from recent clinical trials and basic research show a potential role for SGLT2 inhibitors in treating obesity-associated cardiometabolic complications in PCOS. This narrative review discusses the mechanisms of the beneficial effect of SGLT2 inhibitors in cardiometabolic diseases in PCOS.

## Introduction

Polycystic Ovary Syndrome (PCOS) is the most common endocrine disorder in reproductive-age women, affecting 5-20% of this population (1–3). While there has been evidence of women with PCOS since the time of Hippocrates (4), the syndrome was first described by Stein and Leventhal in 1935 in their article “Amenorrhea associated with bilateral polycystic ovaries” (5). Stein and Leventhal described a series of cases focusing on amenorrhea in women with polycystic ovaries as determined by bimanual pelvic exam and/or pneumoroentgenography (5). However, they also noted physical signs of androgen excess in most cases (5). Since the 1930s, our understanding of the disease has evolved. However, the etiology of PCOS remains unknown, and there are still many gaps in our knowledge and disagreements in the field, in part due to three coexisting sets of criteria for PCOS diagnosis (6–8).

**Figure 1:**
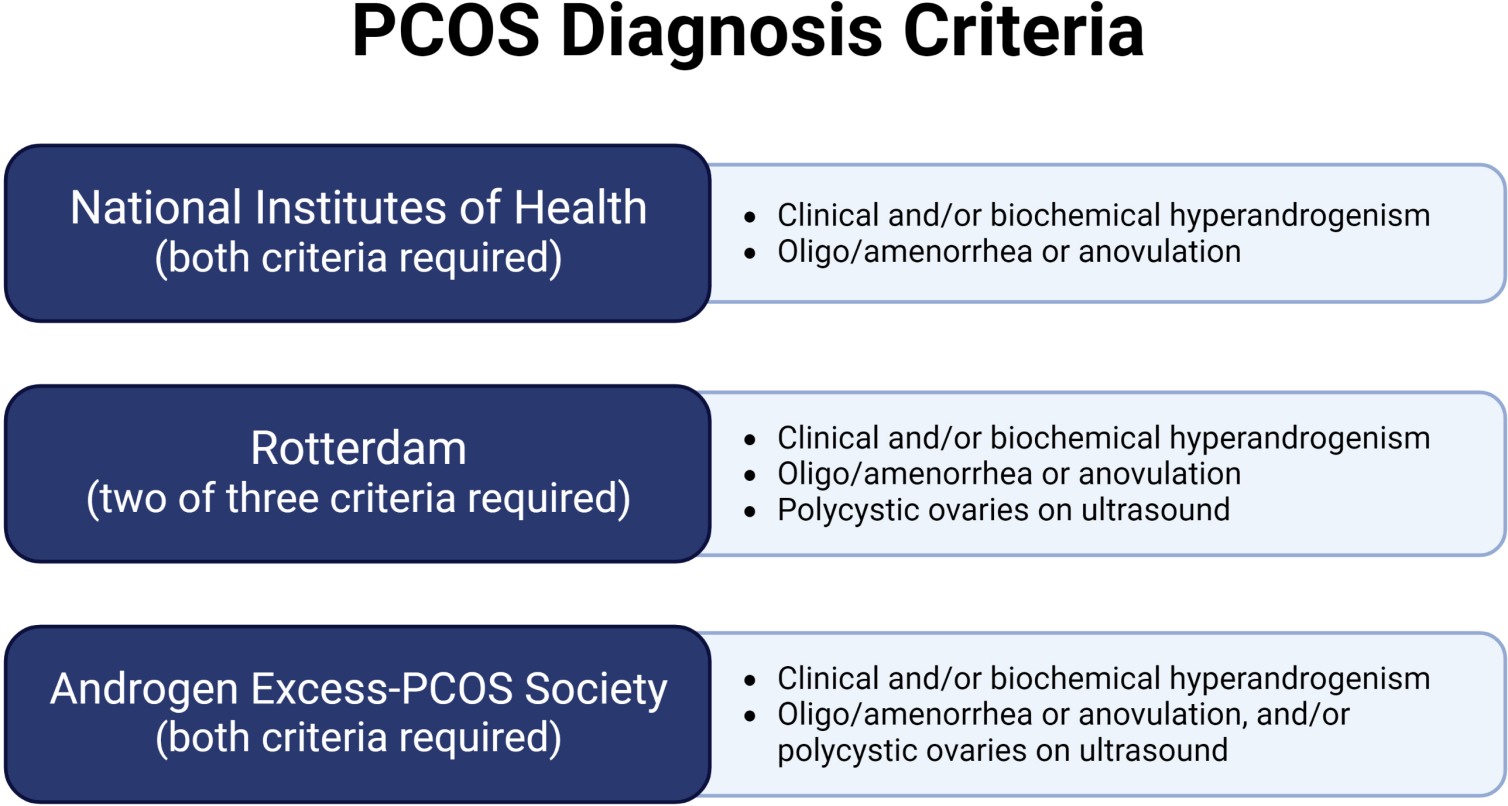
PCOS diagnosis criteria.

PCOS, a diagnosis of exclusion, can be characterized by using three different sets of diagnostic criteria: the National Institutes of Health (NIH) 1990 criteria, the Rotterdam 2003 criteria, and the Androgen Excess-PCOS Society (AE-PCOS) 2006 criteria (6–8) (**Figure 1**). The NIH and AE-PCOS criteria consider androgen excess a requirement for diagnosis. Androgen excess can be defined as either biochemical or clinical evidence of high levels of androgens (6–8). Therefore, if a woman presents to the clinic with hirsutism (as defined by the Ferriman-Gallwey Scale) (9), excessive acne after puberty, or male-pattern balding, then measurement of serum androgens is not necessary for the PCOS diagnosis *per se*. However, biochemical testing, including testosterone levels, is required to eliminate other possible causes of androgen excess, as PCOS is a diagnosis of exclusion. Workup to exclude elevated prolactin, Cushing syndrome, non-classic congenital adrenal hyperplasia, thyroid dysfunction, androgen-producing tumors, and exogenous administration of androgens should be done before conferring the PCOS diagnosis (6–8). In particular, if total serum testosterone is above 200 ng/dL, if dehydroepiandrosterone sulfate is above 800 μg/dL, if signs of excess androgen progress rapidly, or if the voice changes, then the possibility of an androgen-producing tumor in the adrenal gland or ovary should be thoroughly investigated before diagnosing the patient with PCOS (10). Women with PCOS will have elevated levels of androgens, but they should not approach male levels (10, 11). The Rotterdam criteria, the most common criteria used in the clinic, requires the presence of two of the three PCOS’ characteristics (**Figure 1**) generating 4 different phenotypes. As a result, there are women diagnosed with PCOS with and without hyperandrogenism. In recent years, several lines of evidence suggest that women with hyperandrogenic PCOS have worsened cardiovascular profiles (12). Unfortunately, there are no safe and effective therapeutic agents to decrease the levels or block the action of androgens in women. Furthermore, the heterogeneity of the clinical manifestation of PCOS suggests that the involvement of multiple pathophysiological pathways as suggested by GWAS studies (13, 14). Thereby, novel and effective therapeutic agents are needed for safe and effective PCOS clinical management.

Sodium-glucose cotransporter-2 (SGLT2) inhibitors provide cardiovascular protection in patients with and without type 2 diabetes mellitus (T2DM). Although the exact mechanisms of how SGLT2 inhibitors confer cardiovascular protection remains unclear, numerous mechanistic hypotheses have been postulated, including modulation of the renin-angiotensin system and/or the sympathetic nervous system and improvement in mitochondrial function. Data from recent clinical trials and basic research show a potential role for SGLT2 inhibitors in treating obesity-associated cardiometabolic complications targeting those abnormal pathophysiological systems in PCOS. This narrative review discusses the mechanisms of the beneficial effect of SGLT2 inhibitors in cardiometabolic diseases in PCOS.

## Cardiovascular disease in PCOS

Cardiovascular disease is the number one cause of death among females in the United States (15). Unfortunately, PCOS is associated with multiple cardiovascular risk factors, such as obesity, hypertension, insulin resistance, dyslipidemia, and renal injury (16–19). While it is clear that women with PCOS have increased prevalence of cardiovascular risk factors, there is debate about whether or not these cardiovascular risk factors translate into increased cardiovascular events (20). In 2006, a study including ~11,000 women with PCOS from California (USA) showed no increased risk of coronary artery disease, cerebrovascular disease, or peripheral arterial disease in PCOS (17). Later, in 2015, a study including ~20,000 Danish women with PCOS also showed no increased risk of cardiovascular disease (21). However, when the same Danish population was reanalyzed broadening the definition of CVD to include hypertension and dyslipidemia, women with PCOS showed a ~2-fold increase in CVD events (22). The negative findings of the American and some of the Danish studies were in spite of both studies showing increased prevalence of cardiovascular risk factors, such as hypertension, dyslipidemia, and T2DM, in women with PCOS (17, 21). Conversely, in 2020, a meta-analysis showed that women with PCOS have an increased risk of cardiovascular disease and stroke (23). Furthermore, in 2021, a study including ~175,000 British women with PCOS demonstrated an increased risk for cardiovascular events, including myocardial infarction, angina, and revascularization in young women with PCOS (24). Participants were matched with controls for body mass index (BMI) on a 1:1 ratio in a total of 350,000 women, giving unprecedented strength to the work of Berni et al. (20, 24). Although there are multiple pharmacological agents used to manage the cardiometabolic complications in PCOS, their safety and effectiveness to prevent or ameliorate cardiovascular disease and mortality in PCOS are limited (25).

**Figure 2:**
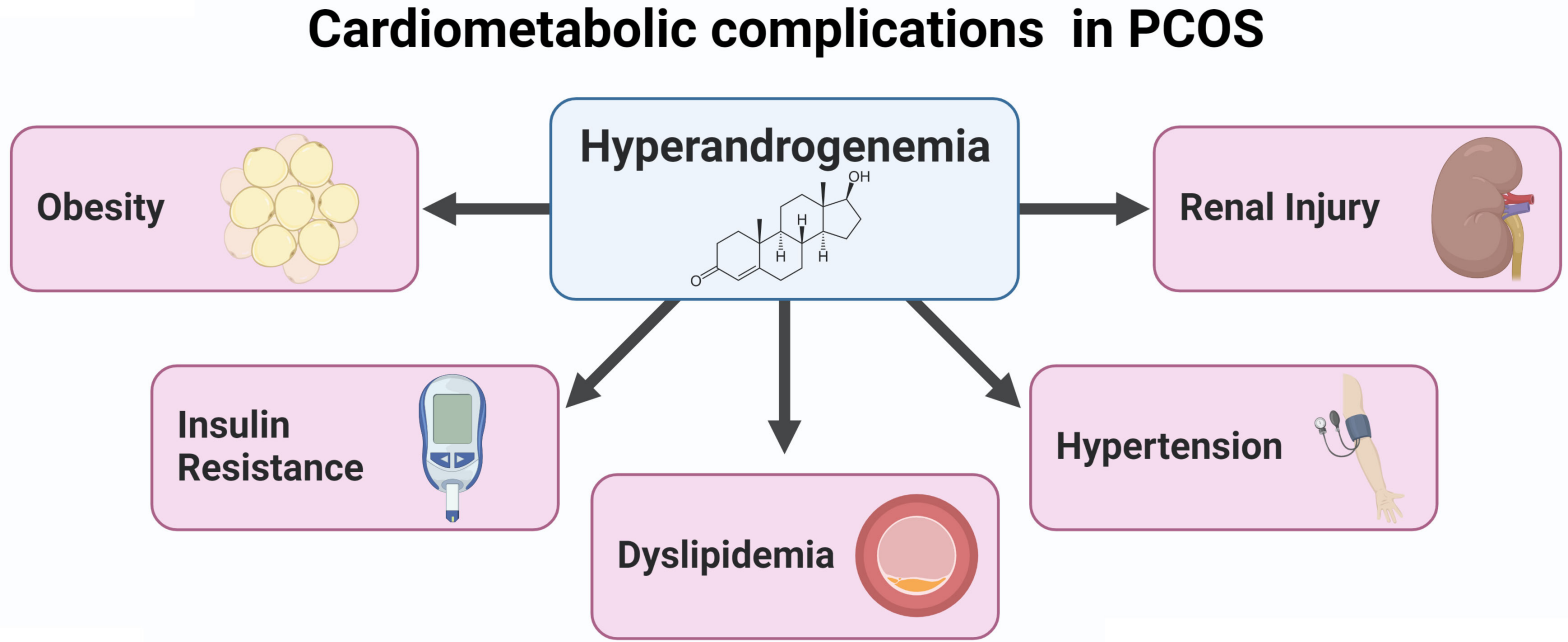
Cardiometabolic complications in PCOS.

Although the etiology of the syndrome remains unknown, hyperandrogenemia may constitute a key mechanism underlying the cardiovascular risk factors in PCOS. We have demonstrated that hyperandrogenemia in female rats elicits several cardiovascular risk factors also present in women with PCOS (**Figure 2**). More recently, we demonstrated the potential benefit of SGLT2 inhibitors in body composition and blood pressure in such PCOS experimental model. Therefore, novel pharmacotherapies, such as sodium-glucose cotransporter-2 inhibitors, could simultaneously target multiple mechanisms of the pathophysiology of the cardiometabolic complications associated with PCOS.

## SGLT2 inhibitors: Discovery and use beyond type 2 diabetes mellitus

### Discovery of SGLT2 and its function

As the kidney filters blood, it must exercise precise, selective control over the reabsorption of electrolytes, carbohydrates, and amino acids while excreting waste products like urea. In the 1930s, with experiments from Shannon and Fisher, glucose in the mammalian nephron was shown to go “into reversible combination with some element in the tubule cells, present in constant but limited amount,” and this mystery element was proposed to be the rate-limiting step of glucose reabsorption from the nephron (26). In the 1970s, Scriver et al. hypothesized that there might be a G1/G2 system to reabsorb hexoses in the kidney, with G1 having a low-capacity, and low affinity for glucose. In contrast, G2 would have a high-capacity, high affinity. Inspired by familial renal glucosuria, Scriver et al. also hypothesized that only the G1 system was present in the intestines while G2 was not (27). Experiments from Turner and Moran in the 1980s gave further evidence for two different sodium-dependent glucose transporters in the proximal tubule of the nephron (28). The G1 and G2 systems were later characterized as low-capacity, high-affinity, or high-capacity, low-affinity and were recognized as SGLT1 and SGLT2, respectively (29). SGLT2 expression was localized to the renal cortex, while SGLT1 was localized to the renal medulla and intestine (29, 30). As SGLT2 has a high capacity for glucose, it was later confirmed that SGLT2 is explicitly responsible for the vast majority of renal glucose reabsorption (31).

### Discovery of SGLT2 inhibitors

By inhibiting SGLT2, blood glucose could be lowered independently of insulin *via* its glucosuric effect, thus minimizing the risk of hypoglycemia observed with other antidiabetic agents (32). However, there were multiple drawbacks to using phlorizin, an early SGLT2 inhibitor naturally found in apple trees (33, 34), such as poor absorption in the gut and its concurrent inhibition of SGLT1 (35). Lack of functioning SGLT1 can lead to diarrhea, as seen with hereditary glucose/galactose malabsorption, so the search began for selective and orally available drugs to inhibit SGLT2 (36). Out of this search came the burgeoning drug class of gliflozins, which were based on the structure of the o-glucoside phlorizin (37, 38). While some gliflozins, such as the o-glucoside sergliflozin, were not successful candidates for managing glucose homeostasis (37), many c-glucosides made it through phase III clinical trials, such as empagliflozin, dapagliflozin, and canagliflozin, which are the SGLT2 inhibitors that are widely used in clinical practice nowadays (38, 39).

### SGLT2 inhibitors confer cardiovascular protection in patients with and without diabetes

Around the time that currently available SGLT2 inhibitors were progressing through clinical trials, the United States Food and Drug Administration (FDA) issued an additional requirement for phase III clinical trials of antidiabetic medications for T2DM (40). Because of evidence that thiazolidinediones, PPARγ agonists that act as insulin sensitizers (41), may increase cardiovascular risk in patients with T2DM even while improving glycemic control, the FDA began recommending that all new antidiabetic medications also needed to demonstrate cardiovascular safety (40, 42).

Intriguingly, with the landmark EMPA-REG study (43), empagliflozin was shown to reduce the risk of cardiovascular death in T2DM, becoming the first antidiabetic medicine proven to do so (40, 43). Additionally, EMPA-REG showed preservation of renal function with empagliflozin (44). Later with the CANVAS (45), DECLARE-TIMI 58 (46), and CREDENCE (47) trials, the SGLT2 inhibitors canagliflozin and dapagliflozin were also shown to decrease the risk of cardiovascular death and renal failure. These benefits have been proposed to be independent of changes in glycemic status (47) as the reduction in blood glucose with SGLT2 inhibition is modest (43, 45, 47), suggesting that the positive benefit of SGLT2 inhibitors could be possible in patients without overt T2DM. Additionally, in terms of side effects, there was no increased risk of hypoglycemia or acute kidney injury with SGLT2 inhibition (43, 45–47). However, there are some notable side effects of this drug class. SGLT2 inhibition does increase the risk of mycotic genital infection (43, 45–47). More rarely, SGLT2 inhibition also increases the risk of diabetic ketoacidosis (46, 47), in particular euglycemic diabetic ketoacidosis (48), often occurring with surgery or illness. Euglycemic diabetic ketoacidosis is a severe and life-threatening complication that can be overlooked by providers because of the normal range blood glucose in this condition, so this is an important consideration for anyone taking or prescribing gliflozins.

More recently, with multiple trials such as EMPEROR-Reduced (49), EMPEROR-Preserved (50), DAPA-HF (51), and DAPA-CKD (52), even in the absence of T2DM, SGLT2 inhibition benefited patients with chronic kidney disease and patients with heart failure (with either reduced or preserved ejection fraction). These findings implicated a potential role of SGLT2 inhibition in cardiovascular or renal disease for various conditions. However, exactly how SGLT2 inhibition produces this cardiovascular/renal protection independently of its effect on glycemia is uncertain and is an open question. There are multiple hypotheses concerning these mechanisms (53, 54).

## SGLT2 inhibition in women with PCOS

**Figure 3:**
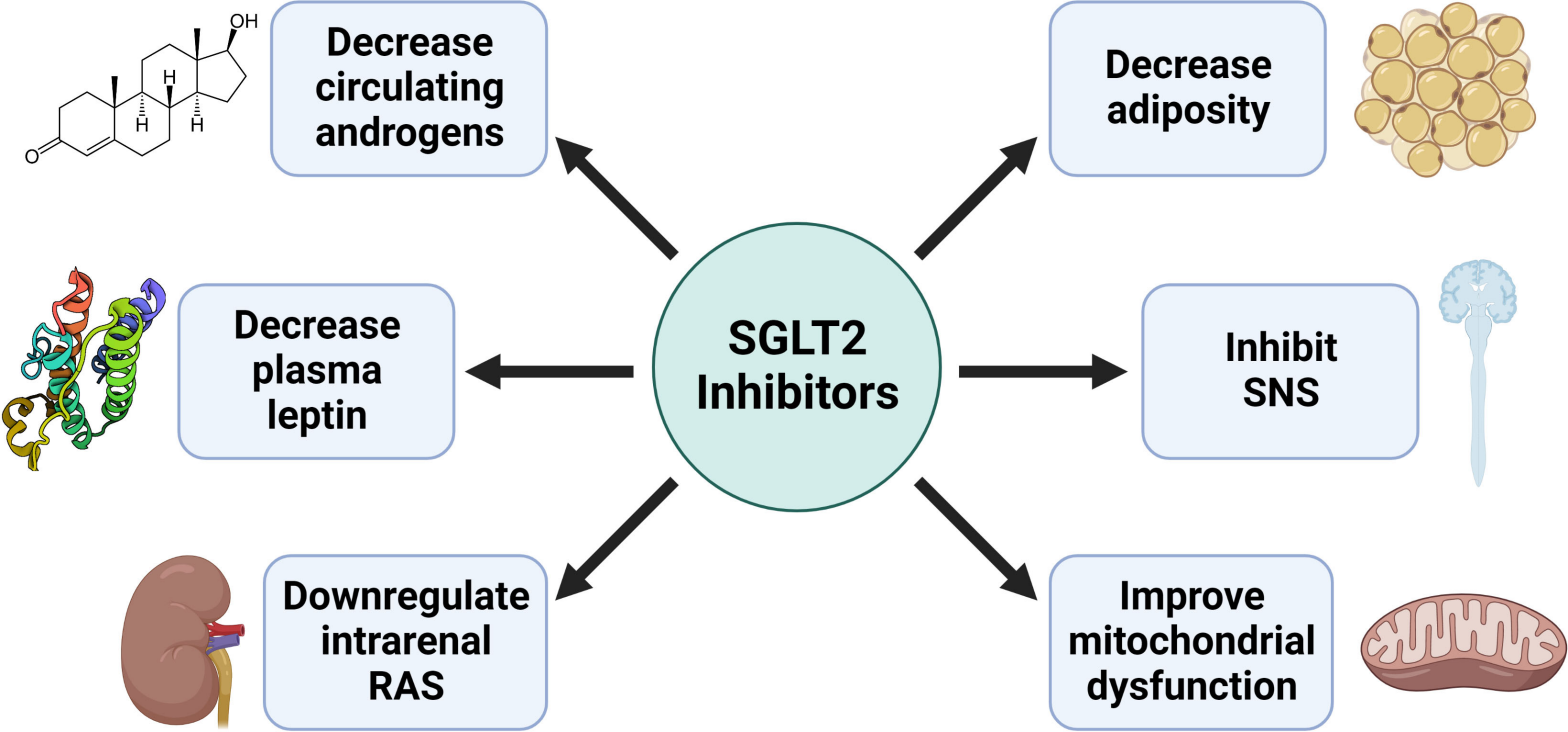
Potential mechanisms by which SGLT2 inhibitors improve the cardiometabolic complications in PCOS.

PCOS is associated with insulin resistance, obesity, renal injury, mitochondrial dysfunction, and activation of both the Sympathetic Nervous System (SNS) and Renin-Angiotensin System (RAS). SGLT2 inhibitors have demonstrated improvements in all these disease states, suggesting they may be a promising novel therapy to improve women’s healthcare in PCOS (**Figure 3**). Recently three small clinical trials used SGLT2 inhibitors to improve the cardiometabolic complications of patients with PCOS with exciting results (see summary in **Table 1**). In the trial by Javed et al., empagliflozin decreased body weight, body mass index, and fat mass in overweight women with PCOS compared to metformin (55). However, there was no decrease in insulin resistance or blood pressure, though it should be noted that the patients in this study had normal blood pressure at baseline (55). In the trial by Elkind-Hirsch et al., they explored if there were a synergistic effect between SGLT2 inhibition with dapagliflozin and glucagon-like peptide-1 receptor agonism (GLP-1RA) with exenatide in obese women with PCOS (56). Their data show that dapagliflozin and exenatide have an additive effect to further reduce body weight and fat mass than either drug individually can, which is likely due to their differing mechanisms of action. Combination therapy of SGLT2 and GLP-1RA could constitute a promising therapeutic tool to ameliorate cardiometabolic complications in PCOS women.

**Table 1:** Clinical trials of SGLT2 inhibitors in PCOS.

Trial	SGLT2 Inhibitor	Number of Participants	Duration of Trial	Findings
Javed et al. 2019 (55)	Empagliflozin	39	12 weeks	↓ Body weight, BMI, and fat mass
Elkind-Hirsch et al. 2021 (56)	Dapagliflozin	92	24 weeks	Additive effect in ↓ body weight and fat mass with GLP-1RA
Tan et al. 2021 (57)	Licogliflozin	20	2 weeks	↓ Insulin Resistance and DHEAS

↓, decrease.

More recently, Tan et al. reported that licogliflozin decreased insulin resistance and circulating dehydroepiandrosterone sulfate (DHEAS) in women with PCOS, with a similar tendency in other circulating androgens (57). However, licogliflozin is not yet approved by the FDA and is more promiscuous than other SGLT2 inhibitors, having only a 30-fold selectivity for SGLT2 over SGLT1 (58), instead of the over 2,500-fold selectivity that empagliflozin has for SGLT2 (38). Higher-quality clinical trials are needed to better define the potential role of selective SGLT2 inhibitors in treating the cardiometabolic complications in women with PCOS.

## Insulin resistance and SGLT2 inhibition in PCOS

While not part of the diagnostic criteria for PCOS, insulin resistance is frequently present in lean and obese women with PCOS (16, 59). In PCOS patients using the sequential euglycemic insulin clamp technique, insulin infusion leads to elevations in testosterone (60). Mechanistically, insulin and insulin-like growth factors synergize with luteinizing hormone to increase androgen synthesis in ovarian theca cells (61). Furthermore, insulin decreases circulating sex hormone-binding globulin (SHBG) by decreasing its hepatic synthesis, leading to increased circulating free testosterone (61), which can bind and activate the androgen receptor. Moreover, testosterone can be reduced to 5α-dihydrotestosterone (DHT) in peripheral tissues to become the most potent endogenous agonist of the androgen receptor (62). However, short-term androgen administration in women also decreases insulin sensitivity, as demonstrated by both hyperglycemic and euglycemic hyperinsulinemic clamps (63). Furthermore, androgen administration in female rats leads to insulin resistance (64, 65). Thus, it is still unclear if insulin resistance causes androgen excess in women with PCOS or whether androgen excess leads to the constellation of cardiometabolic dysfunctions, including insulin resistance, in women with PCOS.

To ameliorate insulin resistance in PCOS, the first-line therapy for T2DM, metformin, is often used off-label (66, 67). However, there is an ongoing debate whether or not metformin is beneficial for insulin resistance in PCOS patients, as a recent meta-analysis showed that metformin had no impact on fasting blood glucose or insulin in overweight women with PCOS (68, 69). Small short-term clinical trials have shown that the FDA-approved anti-obesity glucagon-like peptide-1 receptor agonists (GLP-1 RAs) improve insulin resistance in PCOS patients (70) and may be superior to metformin (71). Therefore, there is a great need for new therapeutics targeting insulin resistance and T2DM in PCOS.

We previously demonstrated that hyperandrogenic female (HAF) experimental PCOS model generated with female Sprague Dawley rats chronically implanted with subcutaneous DHT pellets (7.5 mg/90 days) exhibit similar cardiometabolic complications to those in women with PCOS. The HAF rats exhibit increased food intake, obesity with an expansion of both subcutaneous and visceral fat depots, insulin resistance, and elevated blood pressure, that closely mimic the cardiometabolic complications in women with PCOS (65, 72). This review will focus on the HAF cardiometabolic complications; however, there are multiple animal experimental models of PCOS, each one with pros and cons to model the human disease as summarized in recent reviews (73, 74).

We recently demonstrated that SGLT2 inhibition did not improve insulin resistance in the HAF rats, but it did improve adiposity and elevated blood pressure, suggesting that this agent could exhibit cardioprotective effects in women with PCOS. Whether SGLT2 inhibitors could be used to prevent the progression of insulin resistance to diabetes in women with PCOS remains unknown.

## Hypertension and SGLT2 inhibition in PCOS

### The systemic renin-angiotensin system

Multiple studies have demonstrated an increased prevalence of hypertension in women with PCOS (17, 18). Moreover, blood pressure could be only mildly elevated as demonstrated in 24-hour ambulatory blood pressure measurements in women with PCOS (75). Obesity is associated with hypertension, but women with PCOS, even with normal weight, can have elevated blood pressure (76). The specific mechanism(s) that lead to elevated blood pressure or hypertension in PCOS remain a matter of debate.

One promising mechanism is dysregulation of the renin-angiotensin system (77, 78). The RAS plays a significant role in long-term blood pressure control, and it may lead to hypertension when inappropriately activated (79). The systemic RAS begins with the macula densa in the distal portion of the nephron (80). When it senses low sodium, the macula densa releases the enzyme renin from juxtaglomerular cells. Renin will then cleave angiotensinogen, released mainly from the liver, into angiotensin I (Ang I). Angiotensin-converting enzyme (ACE), which is expressed primarily in the lung, will then convert Ang I into angiotensin II (Ang II). Then, Ang II can bind to the Ang II Type 1 Receptor (AT1R) or the Ang II Type 2 Receptor (AT2R) (80, 81). Through AT1R, Ang II constricts the efferent arteriole, causing an increase in glomerular filtration rate to increase sodium delivery to the macula densa. Furthermore, Ang II stimulates aldosterone release by the adrenal gland *via* AT1R, which will increase sodium reabsorption by principal cells of the collecting duct of the nephron. Alternatively, *via* AT2R, Ang II causes vasodilation to decrease blood pressure. Furthermore, Ang II can be converted by angiotensin-converting enzyme 2 (ACE2) into the heptapeptide angiotensin (1–7) that reduces blood pressure and insulin resistance *via* the Mas receptor (81, 82).

### The intrarenal renin-angiotensin system

In addition to the systemic RAS, several tissue-specific renin-angiotensin systems exist, such as in the kidneys, heart, and adipose tissue (83). We will focus on the intrarenal RAS for this review as SGLT2, an essential player in this work, is predominantly expressed in the kidney but not in the heart or the adipose tissue (84). In the nephron, AT1R lines the lumen of the proximal tubule, distal tubule, and collecting duct (85) and can stimulate angiotensinogen synthesis in the proximal tubule with Ang II-activation (86). Renin is produced in the proximal tubule (87, 88), distal tubule (89), and principal cells of the collecting duct (89), allowing for angiotensin I to be produced within the nephron. Meanwhile, ACE is expressed along the brush border of the proximal tubule (90) and within cells of the collecting duct (91), allowing for Ang I to be produced within the nephron. When Ang II binds to the AT1R of the principal cells of the collecting duct, it increases the activity of epithelial sodium channels, which can further increase blood pressure (86, 91, 92). The detailed mechanism of the regulation of blood pressure by epithelial sodium channels can be found elsewhere (93). Thus, the intrarenal RAS appears to have a positive feedback loop where intratubular Ang II can lead to the formation of more intratubular Ang II, which can lead to increases in blood pressure (86).

### Hypertension in PCOS: Targeting the renin-angiotensin system

The RAS, a central regulator of blood pressure, is modulated by androgens. Women with PCOS have dysregulation of the RAS with high circulating levels of renin (77, 94), the rate-limiting enzyme of the RAS (95). Plasma ACE2, which converts Ang II into the vasodilator angiotensin (1–7), is also decreased in women with PCOS (94). In a case series of four women with PCOS, treatment with telmisartan, an AT1R blocker, normalized blood pressure, reduced androgen levels, and improved the menstrual cycle (78). In a preclinical hyperandrogenemic female (HAF) rat model of PCOS, mRNA expression of renal angiotensinogen and ACE is increased (65). Furthermore, the ACE inhibitor enalapril reduces blood pressure in aged HAF rats more than in controls (96). However, compensatory alterations in the RAS have been shown in PCOS, as circulating angiotensinogen is decreased in women with PCOS (94). Moreover, intrarenal ACE2 is upregulated while intrarenal renin is downregulated in HAF rat model of PCOS (97). The upregulation of the ACE2 could be a protective mechanism to counteract the activation of the classical arm of the RAS; however, this hypothesis needs to be tested. Altogether, these data implicate the RAS is at least partially responsible for the increased blood pressure observed in PCOS. ACE inhibitors or AT1R blockers are widely used as antihypertensive drugs in the general population. However, due to their potential teratogenic, fetotoxic, and miscarriage-associated risks during pregnancy, ACE inhibitors and AT1R blockers are rarely used in the clinic in PCOS women of reproductive age (98–100). Thereby, agents that impact the RAS safely and effectively are needed to treat hypertension in women with PCOS.

### SGLT2 and the renin-angiotensin system

RAS blockers are part of the standard of care for chronic kidney disease (both with and without diabetes mellitus) and heart failure. In landmark clinical trials showing cardiovascular and renal protection by SGLT2 inhibition, most patients were on some form of RAS blocker at baseline (43, 45–47, 49–52). In other words, the benefit of SGLT2 inhibition in clinical trials typically occurred on a background of RAS blockade. How might RAS blockade be working with SGLT2 inhibition, though?

As SGLT2 inhibition decreases sodium reabsorption in the proximal tubule, one would expect that there would be increased sodium delivery to the macula densa, thus reducing renin release and RAS activation. However, what has been found experimentally is more complex. Concerning the first part of the systemic RAS cascade, in a retrospective analysis of patients with hypertension and T2DM, no significant change was observed in plasma renin activity (PRA) with SGLT2 inhibition (101). Meanwhile, diabetic male mice treated with empagliflozin had decreased PRA; however, empagliflozin caused no change in PRA in control mice (102). Furthermore, a small observational study showed in diabetic patients that PRA was initially increased after one month of SGLT2 inhibition but returned to normal after three months of treatment (103). The variability observed in PRA with SGLT2 inhibition could be partly due to volume contraction from SGLT2 inhibition. The glucosuria from SGLT2 inhibition causes osmotic diuresis, which may decrease extracellular volume to trigger the release of renin (53). However, with time, the elevated antidiuretic hormone can compensate for the decrease in volume from SGLT2 inhibition (104), which may restore renin levels to normal.

Renin is far from the only component of the RAS reported to respond to SGLT2 inhibition, and the results are equally as mixed as those of renin. In a mouse model of T2DM, Woods et al. found that renal cortex angiotensinogen mRNA and protein expression was decreased by SGLT2 inhibition; however, renal ACE and AT1R mRNA expression was unchanged (105). Meanwhile, in a rat model of T2DM, Shin et al. found that AT1R protein expression was decreased in the renal cortex with SGLT2 inhibition (106). Furthermore, in diabetic Dahl salt-sensitive rats, SGLT2 inhibition was shown to work synergistically, instead of additively, with ACE inhibition to reduce blood pressure (107). Meanwhile, Bautista et al. found in male rats that Ang II increases renal SGLT2 independent of blood pressure changes and that inhibiting Ang II formation or AT1R decreases renal SGLT2 (108). Therefore, at least in male rodents, SGLT2 inhibitors and RAS blockade may work together synergistically, which may translate to the importance of patients having both types of pharmacotherapies to treat their cardiometabolic disease.

Recently, we reported that SGLT2 inhibition in HAF rats downregulates intrarenal ACE and AT1R mRNA, which was accompanied by a slight decrease in mean arterial pressure (97). However, intrarenal ACE2 mRNA, which is part of the vasodilatory arm of the RAS, was also downregulated by SGLT2 inhibition in HAF rats (97). If the upregulation of the ACE2 in the kidney is a compensatory mechanism to combat the androgen deleterious effect, one can speculate that due to the beneficial effect of SGLT2 inhibitors, this is not further needed. These data suggest that SGLT2 inhibition could work synergistically with RAS blockers to reduce blood pressure in HAF rats, similar as in male rodents (107).

### Sympathetic nervous system and SGLT2 in PCOS

Another possible mechanism for hypertension in women with PCOS is an activation of the SNS. Using heart rate variability to measure autonomic dysfunction, women with PCOS matched with controls for body mass index and blood pressure have increased SNS activity and decreased parasympathetic activity (109). Furthermore, adrenergic blockade with terazocin and propranolol in young HAF rats effectively decreases blood pressure (110). Renal denervation, which reduces sympathetic activity in the kidney, also reduces blood pressure in HAF rats and in women with PCOS (110, 111). The SNS may be activated in PCOS women and HAF rats because of elevations in the adipokine leptin in the circulation (65, 112). Leptin secretion by the adipose tissue is upregulated in obesity. When leptin is chronically elevated, it increases blood pressure by stimulating the sympathetic nervous system *via* melanocortin 4 receptor (MC4R) in pro-opiomelanocortin (POMC) neurons (113). Activation of the renal SNS can also activate the RAS (113) and promote sodium retention, actions that, if sustained over time, can increase blood pressure. Consequently, it is possible to speculate that adrenergic blockade could reduce RAS activation in PCOS. The detailed mechanism by which MC4R regulates blood pressure has been recently reviewed (114).

Adrenergic blockade is part of the standard of care in particular clinical conditions such as heart failure with reduced ejection fraction (115). To the best of our knowledge, randomized clinical trials with adrenergic blockade have not been yet performed in women with PCOS. However, while used to treat some individuals with essential hypertension, adrenergic blockade is not the gold standard, with some studies showing a lack of cardiovascular protection with this drug class (116, 117). Therefore, while the SNS appears to be upregulated in overweight or obese women with PCOS, whether or not direct adrenergic blockade would be beneficial in attenuating their hypertension is still unclear.

There are some evidence that SGLT2 inhibitors could target the SNS to exhibit their cardioprotective effects. Activation of the SNS leads to vasoconstriction and an increase in heart rate, leading to an increase in blood pressure (80). With the potential volume contraction from the osmotic diuresis caused by SGLT2 inhibition, one would not be surprised to observe a compensatory increase in heart rate. However, data from phase II/III clinical trials in patients with T2DM show that SGLT2 inhibition is associated with a decrease in heart rate (118, 119). Why might that be? A study by Herat and colleagues recently demonstrated in Schlager mice, a model of neurogenic hypertension with sympathetic activation, that SGLT2 inhibition decreases SNS innervation of the kidney, accompanied by a reduction of renal norepinephrine (120). However, precisely how SGLT2 regulates renal SNS is uncertain. A recent meta-analysis in patients with T2DM demonstrated that SGLT2 inhibition was associated with decreased circulating leptin (121), and we recently found that SGLT2 inhibition decreases plasma leptin in HAF rats (97). As leptin is known to stimulate the renal SNS, reduced circulating leptin may contribute to how SGLT2 regulates the renal SNS.

## Mitochondrial dysfunction and SGLT2 inhibitors in PCOS

The mitochondrion is an essential organelle for eukaryotic organisms. It is known as “the powerhouse of the cell” because it conducts oxidative phosphorylation, a process necessary to generate enough energy for complex organisms to function (80). Mitochondrial dysfunction can be defined as when mitochondria cannot provide ATP for the cell while minimizing an overflow of naturally formed reactive oxygen species (ROS) from damaging the rest of the cell (122, 123). Methods to measure ROS in humans or animals include assessing total antioxidant capacity in serum or measuring markers of oxidative damage, such as lipid peroxidation, through 2-thiobarbituric acid reactive substances assay (124). Mitochondrial dysfunction has been linked to diabetes (122, 125), metabolic syndrome (125), heart failure (126), chronic kidney disease (127), and PCOS (128).

Women with PCOS have decreased mitochondrial DNA, a marker of mitochondrial content or volume, in circulating leukocytes (129, 130). Decreased inner mitochondrial membrane potential, altered mitochondrial structure, and increased ROS have also been demonstrated in the oocytes of a PCOS mouse model (131). Furthermore, lean women with PCOS have decreased circulating total antioxidant capacity and increased malondialdehyde, indicating increased oxidative stress (59). The mitochondrion is an organizing center for cellular metabolism (80), so there are significant implications for insulin resistance and obesity in PCOS. Additionally, excess oxidative stress can lead to inflammation, further worsening insulin resistance, obesity, and blood pressure in PCOS (132, 133). Therefore, targeting mitochondrial dysfunction may be a promising therapeutic avenue in patients with PCOS. Yilmaz et al. demonstrated in lean women with PCOS that rosiglitazone, a peroxisome proliferator-activated receptor-γ (PPARγ) agonist that stimulates mitochondrial biogenesis (41), increases circulating total antioxidant capacity, decreases circulating malondialdehyde, and decreases insulin resistance (59). However, PPARγ has multiple functions outside of stimulating mitochondrial biogenesis (134), so it is uncertain if specifically increasing mitochondrial content improves these parameters in women with PCOS. Furthermore, body mass index increases with the thiazolidinedione rosiglitazone in women with PCOS (59). However, there are concerns about thiazolidinediones and cardiovascular risk (42), limiting excitement for using PPARγ agonists in women with PCOS. Exploring the potential role of other pharmacotherapeutics that improve mitochondrial function, such as SGLT2 inhibitors (105, 135–138), is a promising new direction in the field.

SGLT2 inhibition has been found to improve mitochondrial function in various ways. In the heart of male rodents with T2DM, SGLT2 inhibition decreases ROS production (135) and also increases the expression of nuclear respiratory factor 1 (NRF1) and PPARγ coactivator 1-α (PGC1α) (136), which positively regulate mitochondrial biogenesis (134). SGLT2 inhibition also decreases markers of oxidative stress in the blood while increasing the activity of the antioxidant enzyme superoxide dismutase (136). Meanwhile, in the kidney of male rodents with T2DM, SGLT2 inhibition normalizes mitochondrial morphology (preventing mitochondria from becoming excessively round or fragmented) while decreasing urinary 8-isoprostane and 8-hydroxydeoxyguanosine, which are markers of oxidative stress (105, 137). In white adipose tissue of male rodents with T2DM, SGLT2 inhibition similarly increased the expression of NRF1 and PGC1α as it did in the heart, which was associated with an increase in mitochondrial DNA, a marker of mitochondrial content (136, 138). The diseases currently indicated for SGLT2 inhibitor use, such as T2DM, heart failure, and chronic kidney disease, have been linked to mitochondrial dysfunction (125–127), so improving mitochondrial function may be an essential pathway for the beneficial effects seen in patients on SGLT2 inhibitors. However, more studies are needed to explore the therapeutic potential of SGLT2 inhibition on mitochondrial dysfunction in women, especially those with PCOS. We recently demonstrated that hyperandrogenemia in the HAF rat model of PCOS causes the expansion of white adipose tissue, which is associated with decreases in mitochondrial content and function in both subcutaneous and visceral adipose tissue (139). Treatment with SGLT2 inhibitors increased the frequency of small adipocytes in visceral adipose tissue without affecting mitochondrial dysfunction in white adipose tissue, oxidative stress, or insulin resistance in the HAF rat model (139). Our study suggests that targeting mitochondrial dysfunction in PCOS may be necessary to improve insulin resistance and that hyperandrogenemia blunts the beneficial effect of SGLT2 inhibitors in the HAF rat model of PCOS.

## Perspective and clinical implications

PCOS is the most common endocrine disorder in reproductive-age women (1–3). Patients with PCOS have an increased incidence of major adverse cardiovascular events (24), likely driven by the increased incidence of cardiovascular risk factors in this population, such as hypertension, insulin resistance, renal injury, and obesity (16–19) (**Figure 2**). Unfortunately, there is a lack of effective, evidence-based pharmacotherapeutics targeted at cardiometabolic disease (25). Meanwhile, SGLT2 inhibitors have been rapidly expanding their clinical indications because of their cardiovascular protection in patients with and without T2DM (43, 47, 51, 52). However, whether and exactly how SGLT2 inhibitors confer cardiovascular protection in PCOS women, with and without diabetes remains to be elucidated pending high quality large clinical trials. Limited clinical data have suggested that women with PCOS have renal and cardiac target organ injury (140). Moreover, a recent study has shown that women with PCOS have a higher risk of preeclampsia/eclampsia, peripartum cardiomyopathy, and heart failure during hospitalizations for delivery (141). Women with PCOS who have increased cardiovascular risk factors as the conditions mentioned above could benefit from SGLT2 inhibitors, pending confirmation with clinical trials. The numerous mechanistic hypotheses for cardiovascular protection include deactivation of the RAS and/or the SNS as well as improvement in mitochondrial function, all of which are abnormal in women with PCOS. Data from recent small clinical trials and basic research show promise for SGLT2 inhibitors in treating some of the cardiometabolic complications in PCOS.

## Author contributions

JP and LY drafted the manuscript. JP, DR, and LY reviewed and edited the manuscript. All authors contributed to the article and approved the submitted version.
